# Health risks for children exercising in an air-polluted environment can be reduced by monitoring air quality with low-cost particle sensors

**DOI:** 10.1038/s41598-023-45426-3

**Published:** 2023-10-25

**Authors:** Zenon Nieckarz, Krzysztof Pawlak, Jerzy A. Zoladz

**Affiliations:** 1 Marian Smoluchowski Institute of Physics, Jagiellonian University, ul. Łojasiewicza 11, 30-348 Kraków, Poland; 2 Department of Zoology and Animal Welfare, Faculty of Animal Science, Agricultural University of Cracow, Kraków, Poland; 3 Chair of Exercise Physiology and Muscle Bioenergetics, Faculty of Health Sciences, Jagiellonian University Medical College, ul. Skawińska 8, 31-066 Kraków, Poland

**Keywords:** Environmental sciences, Health care, Risk factors, Applied physics

## Abstract

A child’s body is highly sensitive to air quality, especially regarding the concentration of particulate matter (PM). Nevertheless, due to the high cost of precision instruments, measurements of PM concentrations are rarely carried out in school areas where children spend most of their daily time. This paper presents the results of PM measurements made by a validated, low-cost university air pollution measurement system operating in a rural area near schools. An assessment of children’s exposure to PM during school hours (8 a.m.–6 p.m.) at different times of the year was carried out. We show that PM_10_ concentrations in the air, particularly in winter, often exceeded the alert values of 50 µg m^−3^, posing a health risk to children, especially when children exercise outside the school building. We also calculated the rate and total PM_10_ deposition in the respiratory tract during various physical activities performed in clean and polluted air. Monitoring actual PM_10_ concentrations as presented in this paper, using a low cost sensors, offer school authorities and teachers an opportunity to reduce health risks for children. This can be achieved by adjusting the duration and exercise intensity of children’s outdoor physical activities according to the measured air quality.

## Introduction

Protecting the health of the population is an essential policy task for nations^1^, the European Union (EU)^2^, and worldwide^3^. Due to the high sensitivity to the impact of the environment, special attention must be paid to protecting the health of school children^4^. One of the key public health problems is currently exposure to high concentrations of particulate matter in ambient air, both in urban and rural populations.

Low-cost systems for monitoring the concentration of airborne particulate matter are made by government institutions^5^ and also are being developed at universities^6, 7^. Currently, almost every major city (e.g., in the EU^8^) possesses at least one air quality monitoring station provided by the government (state or local government)^5^. Unfortunately, highly accurate systems are too expensive to be purchased with available funds and for the needs of small communities. The use of low-cost measurement networks makes it possible to monitor air quality in smaller towns and villages, thereby reducing the technological exclusion of this mainly rural part of the population (e.g., the rural population comprised 40.1% of Poland’s total population in 2020)^9^.

The negative effects of particulate matter on human health are widely documented, with a particular focus on the harmful effects of the fine particles such as PM_2.5_ and PM_1_ on various tissues/organs of the human body^10, 11^. However, the most commonly measured indicator of air pollution worldwide is the PM_10_. Therefore, in this study we focus on this indicator in terms of its potential impact on children's health. It is worth noting that the particulate matter included in this indicator (PM_10_) also comprises particulate matter labelled as PM_2.5_, PM_1_, etc. For example, the PM_2.5_/PM_10_ ratio over the study area was ~ 0.82, as reported by Wilczyńska-Michalik and Michalik^12^ and Nieckarz and Zoladz^7^.

In this paper, we highlight a potential health hazard related to the outdoor physical activity of children conducted in polluted air. Results are reported of airborne particulate matter readings using the low-cost Storm&DustNet measurement network^7^. Measurements were taken near schools in several villages in the Małopolska (Lesser Poland) Province in Poland, and the degree of potential hazard to schoolchildren from the observed polluted air was assessed.

## Materials and methods

### Characteristics of measurements stations

In the present study, we measure air pollution, air temperature (T), humidity (H), and pressure (PR) utilizing university measuring stations (UMS). These stations belong to a low-cost air monitoring system that is part of the Storm&DustNet scientific project of Jagiellonian University in Kraków, Poland^7^. The UMS continuously measure the airborne mass concentration of particulate matter (PM), namely PM_1_, PM_2.5_, and PM_10_, and the concentration of suspended particulate matter (C) in five diameter ranges (0.3–0.5 µm, 0.5–1.0 µm, 1.0–2.5 µm, 2.5–5.0 µm, 5.0–10.0 µm). Samples are taken 30 times per minute, accumulated to obtain average values per minute, and transferred to a database server by wireless GSM technology. Finally, we analyzed the average values of concentrations calculated based on stored 1-min data. The UMS measure mass concentration with a precision of ± 9 µg·m^−3^ in a wide range of data (from a few up to 240 µg·m^−3^), while the levels of temperature, humidity, and pressure precision are ± 1 °C, ± 3% RH, and ± 1 hPa, respectively^13^.

### UMS locations

Eleven UMS stations were mounted on buildings at a height of approximately 3 m above ground level. The UMS (labeled by letters: A, B, C, D, E, F, G, H, I, J, K) are distributed over the Małopolska Province in southern Poland. All selected places were away from highways and roads with heavy traffic. The study area was contained within a 6 × 10-km rectangle, with distances between stations ranging from 1.5 to 4 km. Particulate matter measurements were carried out over a period from 1 September 2018 to 31 August 2022 (1461 days) covering four heating periods in 11 locations. Station K was installed in a country town close to a primary school. The next eight stations were installed in villages close to primary schools (stations: A, B, D, E, F, G, H, I), and one was placed close to a nursery school (station C). An additional station (J) was installed as a background station in a village with a small population close to green areas where the building density was low.

## Results

### Dust hazards in the studied locations as places in everyday life

The analysis was carried out for both the entire 4-year period considered (1 September 2018 to 31 August 2022) and also for two separate periods: “cold” from October to March (X–III) and “warm” from April to September (IV–IX).

The highest values of average PM_10_ concentration over the overall period considered were recorded by stations K and A, equaling 43.1 µg·m^−3^ and 42.4 µg·m^−3^, respectively. Similarly, the highest mean concentration was recorded during the cold period, equaling 65.4 µg·m^−3^ and 65.3 µg·m^−3^ for stations K and A, respectively. The highest daily PM_10_ concentration occurred on 3 January 2021 in station A (328.8 µg·m^−3^).

On the other hand, throughout the period under review, the lowest average PM_10_ values were recorded by stations I and J, amounting to 28.7 and 29.0 µg·m^−3^, respectively. In all locations, the average PM_10_ value in the warm period did not exceed level 19 µg·m^−3^ (see Table 1).

**Table 1:** Average values of PM_10_ for the warm and cold periods (April–September and October–March), respectively, and for the entire period of the measurements considered in this study (i.e., from 1 September 2018 to 31 August 2022).

Localization	Average PM_10_ for total time [µg·m^−3^]	Average PM_10_ for warm period [µg·m^−3^]	Average PM_10_ for cold period [µg·m^−3^]	Ratio PM_10_ (cold) to PM_10_ (warm)	Date of the daily PM_10_ maximum occurrence	Maximal value of the daily PM_10_ during the study period [µg·m^−3^]
A—village	42.4	15.3	65.3	4.27	03.01.2021	328.8
B—village	30.9	16.3	44.6	2.74	29.12.2021	207.7
C—village	32.9	14.3	51.2	3.58	21.01.2019	234.8
D—village	39.2	16.6	62.2	3.75	21.01.2019	289.1
E—village	36.3	15.7	54.2	3.45	21.12.2020	229.1
F—village	33.0	13.7	51.7	3.77	29.12.2021	267.0
G—village	35.4	15.2	52.0	3.42	21.01.2019	278.7
H—village	36.4	16.6	54.2	3.27	29.12.2021	239.7
I—village	28.7	15.2	42.4	2.79	21.01.2019	234.3
J—village	29.8	18.1	41.8	2.31	22.01.2019	268.9
K—small town	43.1	14.4	65.4	4.54	30.11.2018	302.2
Regional average ± SD	35.3 ± 4.8	15.6 ± 1.3	53.2 ± 8.4	3.4 ± 0.6		261.8 ± 36.3

**Figure 1:**
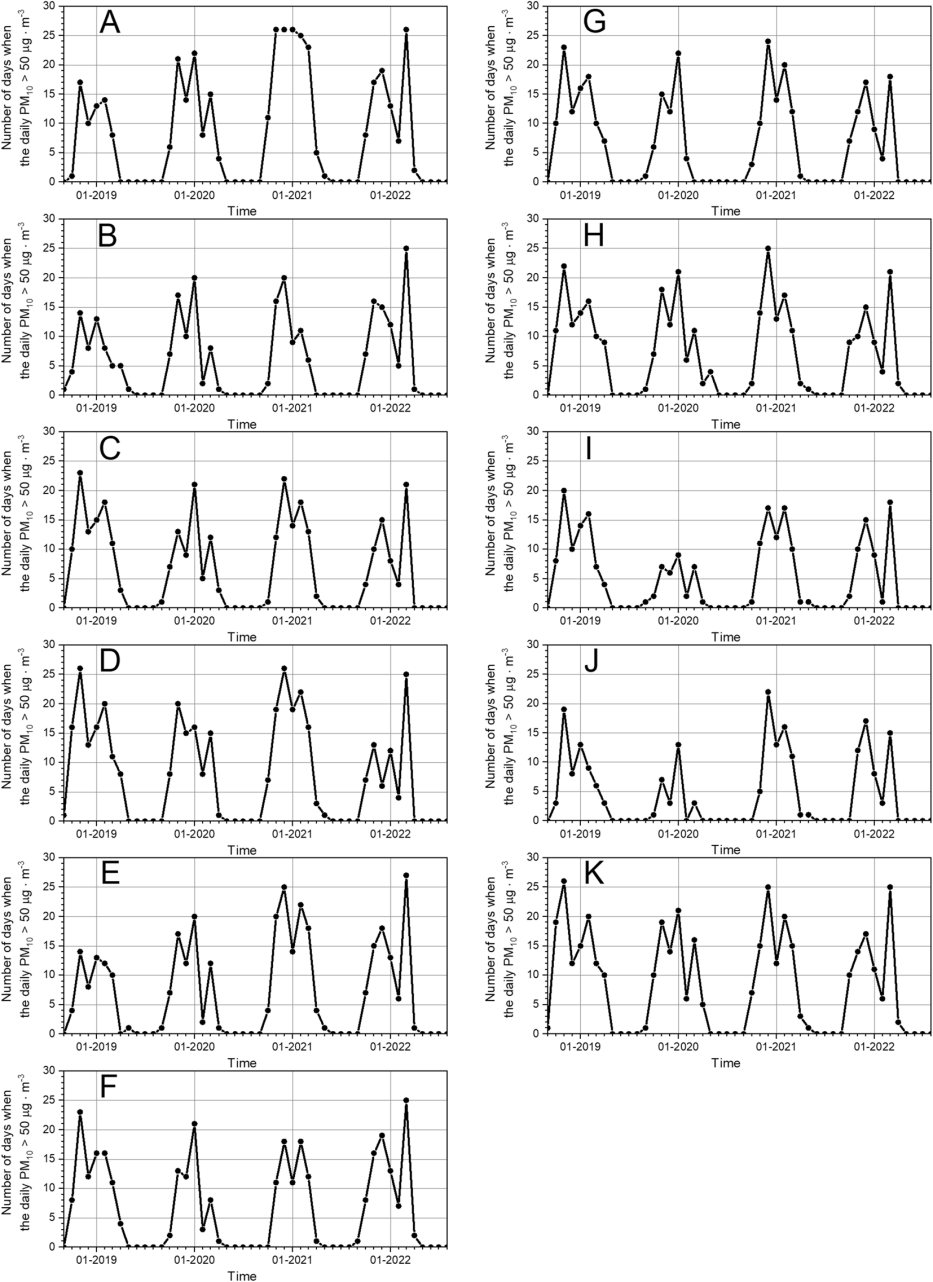
Time distributions of a monthly number of days when the daily PM_10_ exceeded the value 50 µg·m^−3^ recorded by the 11 UMS stations **(**labeled with letters A to K) during the period 1 September 2018 to 31 August 2022.

In the analyzed period, each measuring station recorded several dozen days in each cold period during which PM_10_ concentrations exceeded the permissible level of 50 µg·m^−3^ (see Table 2, Fig. 1). The highest number of days (137) exceeding the permissible levels was recorded by station A in the cold period X 2020–III 2021, while the lowest number of days with the PM_10_ > 50 µg·m^−3^ was recorded by the station J in the cold period X 2019–III 2020.

**Table 2:** Number of days in the cold period when the daily average PM_10_ concentration exceeded 50 µg·m^−3^.

Localization	Cold periods			
	X 2018–III 2019	X 2019–III 2020	X 2020–III 2021	X 2021–III 2022
A—village	63	86	137	90
B—village	52	64	64	80
C—village	90	67	80	62
D—village	102	82	109	67
E—village	61	70	103	86
F—village	86	59	70	88
G—village	89	59	83	67
H—village	85	75	82	68
I—village	75	33	68	55
J—village	58	27	67	55
K—small town	104	86	94	83
Regional average ± SD	79 ± 18	64 ± 20	87 ± 22	73 ± 13

The average number of days exceeding the permissible level of 50 µg·m^−3^ in the cold period for all stations in the analyzed period is 75.7. In period X 2020–III 2021, the highest average number of days exceeding the acceptable level was recorded 87.0; and the smallest (64.4 d) was recorded during the cold period X 2019–III 2020.

In the overall period under review (1 September 2018 to 31 August 2022), the highest number of days amounting to 390 exceedances of the daily permissible level of 50 µg·m^−3^ was reported by station K, and the smallest number of exceedance days (212) was recorded by station J (see Table 3). The maximum number of days exceeding the permissible level in monthly intervals is 27, which was recorded in March 2022 by station E (see Fig. 1).

**Table 3:** Total number of days during which the daily average value of PM_10_ exceeded the levels of 50 µg·m^−3^ and 100 µg·m^−3^, in the period from 1 September 2018 to 31 August 2022.

Localization	Total number of days when PM_10_ concentration exceeded 50 µg m^−3^	Total number of days when PM_10_ concentration exceeded 100 µg m^−3^·
A—village	388	149
B—village	269	50
C—village	308	76
D—village	374	108
E—village	329	81
F—village	312	79
G—village	307	74
H—village	331	100
I—village	239	40
J—village	212	42
K—small town	390	142
Regional average ± SD	314 ± 58	86 ± 37

**Figure 2:**
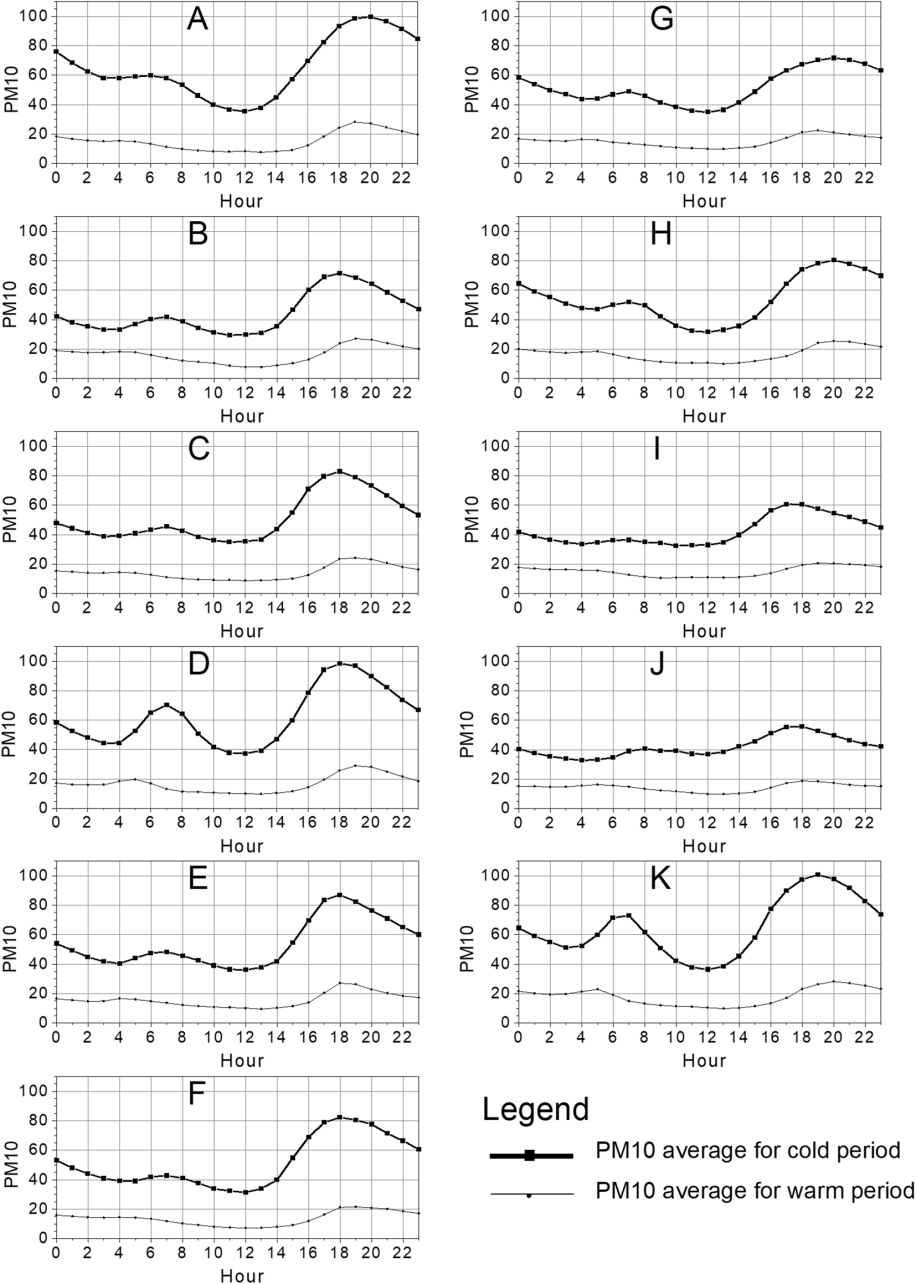
Time distribution of hourly average PM_10_ [µg·m^−3^] concentration during all warm periods (thin line) and the cold period (bold line) recorded by 11 UMS stations **(**labeled with letters A to K).

Average daily distributions of the hourly PM_10_ in all localization are bimodal (Fig. 2). The highest value of PM_10_ was achieved within the hours of 6–8 p.m. The second maximum is much weaker and occurs in the hours 6–8 a.m. On average, in the cold period, PM_10_ is several times higher than in the warm period (see Table 1). Moreover, the largest increase was recorded by station K (4.5), and the smallest increase in the cold period was recorded by station J (2.3).

In almost all locations, the optimal 2-h period with the lowest average PM_10_ concentration occurs between 12 and 2 p.m. except for station I, where this period falls from 11 a.m. to 1 p.m. The overall average value for the cleanest 2-h period across all locations is 34.6 µg m^−3^ (see Table 4), while for the same hours during the warm period, the average value is 10.0 µg m^−3^. Figure 2 shows a noticeable decrease in PM_10_ concentration at all locations around 10 a.m., indicating that the period between 10 a.m. and 2 p.m. has the lowest PM_10_ levels in the study area. This finding is consistent with research conducted by Nieckarz and Zoladz^7^.

**Table 4:** The 2-h periods with the lowest average values of PM_10_ were registered in the cold season (between 8 a.m. and 6 p.m.).

Localization	2-h period in Local Time	Mean value of the PM_10_ for the selected two-hour periods
A—village	12–2 p.m	36.0
B—village	12–2 p.m	29.7
C–village	12–2 p.m	35.2
D–village	12–2 p.m	37.5
E—village	12–2 p.m	36.3
F–village	12–2 p.m	31.9
G–village	12–2 p.m	35.4
H–village	12–2 p.m	31.9
I–village	11 a.m.–1 p.m	32.8
J–village	12–2 p.m	37.1
K–small town	12–2 p.m	37.2
Regional average ± SD		**34.6 ± 2.6**

### Deposition factor (DF)

Based on previous research^16–19^, we assume that the value of the deposition factor at rest (DF_At_ _rest_) and deposition factor for exercising children (DF_Exercise_) are equal to 0.60 and 0.40, respectively, which represents the average mass deposition fraction of PM_10_ in the human respiratory tract. We assume one DF value for boys and girls in all analyzed age groups (from 9 to 16 years).

1 $$TD_{At rest/Exercise} = DF_{At rest/Exercise} \cdot \dot{V}_{E} \cdot TA \cdot PM_{10}$$

where TD represents total deposition of PM_10_ calculated with Eq. (1) (using DF for at-rest and exercise, respectively, according to Table 6) in the volume of ventilated air (minute ventilation V̇_E_) during time activity (TA).

## Discussion

*Air quality in warm and cold periods.* As presented in Table 1, the mean values of PM_10_ in the air in the locations included in this study vary significantly in warm and cold periods. In particular, the PM_10_ concentration in the air in the cold period is 3.4-fold higher than in the warm period (Table 1). Note that the PM_10_ level even in the warm period often exceeds the barrier of 50 µg m^−3^ (Table 3).

*Children’s physical activities* According to the *Physical Activity Guidelines for Americans,* 2nd edition^20^, issued by the U.S. Department of Health and Human Services, the recommended amount of physical activity for children and adolescents at ages 6 through 17 years is 60 min (1 h) or more of moderate-to-vigorous physical activity daily. The statement of this organization underlines the point that regular physical activity in children and adolescents promotes health and fitness. Physically active youth have higher levels of fitness, lower body fat, stronger bones and muscles, and better resilience to stressful situations. In addition, physically active children have better cognitive performance (for a review, see *Physical Activity Guidelines for Americans*, 2nd edition [health.gov]). Healthy children spontaneously undertake various kinds of physical activities such as soccer, football, handball, cycling, or running (for an overview, see Rowland^21^), which often exceeds the above-mentioned recommended 60 min of physical activity.

Interestingly, the endurance capacity of children at age 8–16 years old is remarkably good, as judged based on the levels of their maximal oxygen uptake (V̇O_2max_). For example, Reybrouck et al.^15^ reported the V̇O_2max_ in boys aged 9–16 in the range of 50.6–56.6 mL O_2_ min^−1^ kg^−1^ and in girls at the same age between 42.2 and 43.7 mL O_2_ min^−1^ kg^−1^. Similar values of V̇O_2max_ for the same age groups of children were recently reported by Lai et al.^14^. These findings show that the values of V̇O_2max_ in children, expressed in relative values, are on a level similar to or even higher than healthy adults^22–24^. Moreover, Reybrouck et al.^15^ reported that the oxygen uptake of children at the ventilatory anaerobic threshold at age 9–16 years old amounted on average to 31.7 mL O_2_·min^−1^·kg^−1^ in boys and to 30.4 mL O_2_ min^−1^ kg^−1^ in girls. This result corresponds to about 60% and 65% of their V̇O_2max_, respectively, for girls and boys. Accordingly, it has been reported that prepubertal boys at age 11.6–14 years old could perform an exercise in laboratory conditions (walking/running on a treadmill) lasting 60 min, which required about 60% of their V̇O_2max_ (31.4 − 32.6 mL O_2_ min^−1^ kg^−1^) without symptoms of fatigue (no blood lactate accumulation and submaximal HR during exercise)^25^.

*Physical activity and the minute ventilation.* Any form of sustained physical activity requires an adequate supply of oxygen to the working muscles to generate the needed amount of energy (ATP)^22, 23, 26^. A given metabolic rate (~ V̇O_2_) requires an appropriate minute ventilation (V̇_E_)^22^. The V̇_E_ in children at ages between 9 and 16 years old when at rest amounts to ~ 7–10 L min^−1^, but during maximal exercise, V̇_E_ increases to its maximal values (V̇_Emax_), ranging from ~ 60 to 85 L min^−1^ in girls and from ~ 60 to 115 L min^−1^ in boys depending on their age (see Table 5). The enhanced V̇_E_ during exercise will increase the amount of the various PM inhalation and deposition in the respiratory tract^19^. This issue becomes especially relevant when exercising above the power output corresponding to the change point in V̇O_2_ (~ the lactate threshold)^23, 24, 27^, since above this exercise intensity the V̇_E_ in humans increases non- proportionally to the increase of the exercise intensity^22–24, 27^.

**Table 5:** Value of 100%, 75%, and 40% of V̇_E max_ [L min^−1^] and V_E_ at rest for girls and boys in four age groups.

	AG1	AG2	AG3	AG4
Age [years]	9–10	11–12	13–14	15–16
Girls: V̇_E_ max	57.8	67.6	77.3	85.6
Girls: 75% of V̇_E _max	43.4	50.7	58.0	64.2
Girls: 40% of V̇_E_ max	23.1	27.0	30.9	34.2
Girls: V̇_E_ at rest	7.2	8.6	8.0	8.9
Boys: V̇_E_ max	57.0	73.2	102.7	115.2
Boys: 75% of V̇_E _max	42.8	54.9	77.0	86.4
Boys: 40% of V̇_E_ max	22.8	29.3	41.1	46.1
Boys: V̇_E_ at rest	7.0	8.0	9.0	10.0

Value calculated based on the data collected in a paper by Lai et al.^14^ (for comparisons, see also Reybrouck et al.^15^).

Depending on individual children’s physical capacity, the exercise intensity of physical activities undertaken in the framework of physical education lessons as well as during additional spontaneous physical activities will vary between the children at varied ages. Exercise intensity will influence the magnitude of the absolute V̇_E_ during exercise. In the present study (Table 5) we have presented data of simulations of varied exercise conditions including: (i) heavy–severe physical exercise, such as 1000-m competitive running with the V̇_Emax_, (ii) moderate–heavy intensity exercise with the V̇_E_amounting to 75% V̇_Emax_, and (iii) moderate exercise intensity with the V̇_E_ amounting to 40% V̇_Emax_.

*Air quality and PM*_*10*_* deposition.* As presented in Table 6 and 7 we have calculated the rate (µg min^−1^) and the total PM_10_ (µg) deposition during various physical activities that require different levels of minute ventilation in children (girls and boys) for varied age groups. Note that the rate of deposition during all forms of exercise markedly increases above its levels at rest (see Tables 6 and 7). Regarding exercise, we show data for both variables (i.e., the rate and total PM_10_ deposition) as we believe that these variables should be considered separately. For example, in the case of intensive exercises (e.g., a 1000-m race) frequently practiced during physical education classes in school or other forms of intense exercise will result in a relatively low amount of the total deposition of PM_10_ but a high level of the deposition rate.

**Table 6:** Deposition rate and the total deposition of PM_10_ at rest and during various physical activities (for a description, see the methods section) resulting in different levels of the minute ventilation (V̇_E_) in girls at varied ages (groups AG1–AG4).

Periods: Cold and Warm	Minute ventilation	Time of activity	PM_10_	DF_Atrest/Exercise_	Deposition rate [ug·min^–1^]				Total deposition [ug]			
					AG1	AG2	AG3	AG4	AG1	AG2	AG3	AG4
Calculations made using data from the 2-h periods with the lowest PM_10_ concentrations (12–2 p.m.) during the cold period	Girls: V̇_E_ at rest	1 min	34.6	0.6	0.15	0.18	0.17	0.18	0.15	0.18	0.17	0.18
	Girls: V̇_E_ max	3.5 min	34.6	0.40	0.80	0.94	1.07	1.18	2.8	3.27	3.74	4.15
	Girls: 75% of V̇_E _max	90 min	34.6	0.40	0.60	0.70	0.80	0.89	54.1	63.2	72.2	80
	Girls: 40% of V̇_E_ max	90 min	34.6	0.40	0.32	0.37	0.43	0.47	28.8	33.6	38.5	42.6
Calculations made using data from the 2-h periods with the lowest PM_10_ concentrations (12–2 p.m.) during the warm period	Girls: V̇_E_ at rest	1 min	10.0	0.60	0.04	0.05	0.05	0.05	0.04	0.05	0.05	0.05
	Girls: V̇_E_ max	3.5 min	10.0	0.40	0.23	0.27	0.31	0.34	0.81	0.95	1.08	1.2
	Girls: 75% of V̇_E _max	90 min	10.0	0.40	0.17	0.2	0.23	0.26	15.6	18.3	20.9	23.1
	Girls: 40% of V̇_E_ max	90 min	10.0	0.40	0.09	0.11	0.12	0.14	8.3	9.7	11.1	12.3
Calculations made using data from the 2-h periods with the highest PM_10_ concentrations (4–6 p.m.) during the cold period	Girls: V̇_E_ at rest	1 min	56.7	0.60	0.24	0.29	0.27	0.30	0.24	0.29	0.27	0.30
	Girls: V̇_E_ max	3.5 min	56.7	0.40	1.31	1.53	1.75	1.94	4.59	5.37	6.14	6.79
	Girls: 75% of V̇_E _max	90 min	56.7	0.40	0.98	1.15	1.32	1.46	88.6	103.5	118.4	131
	Girls: 40% of V̇_E_ max	90 min	56.7	0.40	0.52	0.61	0.7	0.78	47.2	55.1	63.1	69.8
Calculations made using data from 2-h periods with the highest PM_10_ concentrations (4 p.m.—6 p.m.) during the warm period	Girls: V̇_E_ at rest	1 min	10.4	0.60	0.04	0.05	0.05	0.06	0.04	0.05	0.05	0.06
	Girls: V̇_E_ max	3.5 min	10.4	0.40	0.24	0.28	0.32	0.36	0.84	0.98	1.13	1.25
	Girls: 75% of V̇_E _max	90 min	10.4	0.40	0.18	0.21	0.24	0.27	16.2	19	21.7	24
	Girls: 40% of V̇_E_ max	90 min	10.4	0.40	0.1	0.11	0.13	0.14	8.6	10.1	11.6	12.8

**Table 7:** Deposition rate and the total deposition of PM_10_ at rest and during various physical activities (for a description, see the methods section) resulting in different levels of the minute ventilation (V̇_E_) in boys at varied ages (groups AG1–AG4).

Periods: cold and warm	Minute ventilation	Time of activity	PM_10_	DF_Atrest/Exercise_	Deposition rate [ug·min^–1^]				Total deposition [ug]			
					AG1	AG2	AG3	AG4	AG1	AG2	AG3	AG4
Calculations made using data from the 2-h periods with the lowest PM_10_ concentrations (12–2 p.m.) during the cold period	Boys: V̇_E_ at rest	1 min	34.6	0.60	0.15	0.17	0.19	0.21	0.15	0.17	0.19	0.21
	Boys: V̇_E_ max	3.5 min	34.6	0.40	0.79	1.01	1.42	1.59	2.76	3.55	4.97	5.58
	Boys: 75% of V̇_E _max	90 min	34.6	0.40	0.59	0.76	1.07	1.2	53.3	68.4	95.9	107.6
	Boys: 40% of V̇_E_ max	90 min	34.6	0.40	0.32	0.41	0.57	0.64	28.4	36.5	51.2	57.4
Calculations made using data from the 2-h periods with the lowest PM_10_ concentrations (12–2 p.m.) during the warm period	Boys: V̇_E_ at rest	1 min	10.0	0.60	0.04	0.05	0.05	0.06	0.04	0.05	0.05	0.06
	Boys: V̇_E_ max	3.5 min	10.0	0.40	0.23	0.29	0.41	0.46	0.8	1.02	1.44	1.61
	Boys: 75% of V̇_E _max	90 min	10.0	0.40	0.17	0.22	0.31	0.35	15.4	19.8	27.7	31.1
	Boys: 40% of V̇_E_ max	90 min	10.0	0.40	0.09	0.12	0.16	0.18	8.2	10.5	14.8	16.6
Calculations made using data from the 2-h periods with the highest PM_10_ concentrations (4–6 p.m.) during the cold period	Boys: V̇_E_ at rest	1 min	56.7	0.60	0.24	0.27	0.31	0.34	0.24	0.27	0.31	0.34
	Boys: V̇_E_ max	3.5 min	56.7	0.40	1.29	1.66	2.33	2.61	4.52	5.81	8.15	9.14
	Boys: 75% of V̇_E _max	90 min	56.7	0.40	0.97	1.25	1.75	1.96	87.4	112.1	157.2	176.4
	Boys: 40% of V̇_E_ max	90 min	56.7	0.40	0.52	0.66	0.93	1.05	46.5	59.8	83.9	94.1
Calculations made using data from the 2-h periods with the highest PM_10_ concentrations (4–6 p.m.) during the warm period	Boys: V̇_E_ at rest	1 min	10.4	0.60	0.04	0.05	0.06	0.06	0.04	0.05	0.06	0.06
	Boys: V̇_E_ max	3.5 min	10.4	0.40	0.24	0.3	0.43	0.48	0.83	1.07	1.5	1.68
	Boys: 75% of V̇_E _max	90 min	10.4	0.40	0.18	0.23	0.32	0.36	16	20.6	28.8	32.3
	Boys: 40% of V̇_E_ max	90 min	10.4	0.40	0.09	0.12	0.17	0.19	8.5	11	15.4	17.3

This scenario is opposite to the situation at rest or during prolonged modern exercise (40% of V̇_Emax_) where the deposition rate is much smaller, but the total deposition is much greater than during the short-term (3.5 min) maximal exercise. It seems to be likely, that the high deposition rate of PM_10_ might have a more acute harmful acute effect on the tissues of the respiratory tract, whereas the high total deposition rate might result in chronic illnesses of the respiratory tract. This hypothesis, however, requires detailed clinical studies in the future. Furthermore, it can be seen in Table 6 that the children at higher ages (see, e.g., groups AG3 and AG4 vs. AG2 and AG1) are exposed to a greater deposition rate and total deposition of PM_10_ as their absolute values of the V̇_Emax_ are much higher than in younger children (see Tables 6 and 7). As shown in Table 1 the sessional and daily changes of the levels of PM_10_ in the inspired air strongly affect the rate and total deposition of PM_10_ in the respiratory tract of children.

*PM*_*10*_* deposition and health risk* The deposited dose of inhaled PM was measured over varied areas (urban, roads, and rural), as well as the dose rates in terms of PM_2.5_ and PM_10_^16–19, 28–32^. These studies indicate that the dose rate was dependent on a few elements, such as geographic factors, physical characteristics of the particle number size distribution, activity type (exercise/at rest), age, gender, concentration metric (number versus mass), and particle diameter.

Studies have shown that the dose rate was nonlinearly proportional to the exposure level. Deep breathing pulls PM faster and farther into the lungs, bypassing initial areas of deposition^33^. According to Ginsberg et al.^34^, the pulmonary region of the lung has slower clearance; therefore, PM remains there longer. Consequently, the particle dose can be two- to four-fold higher among young children. A comprehensive review and description concerning the available models of inhaled particle deposition in the lungs can be found in Morawska et al.^35^. The above-discussed harmful effects of PM on the health of children become particularly relevant when children undertake various forms of physical activities in the polluted air, resulting in an enhancement of the rate and total PM deposition in the respiratory tract (see Tables 6 and 7).

As seen in Tables 6 and 7, the values of the deposition rate and the total deposition for boys from older age groups (AG2–AG4) when exercising at the same percentage of the V̇_Emax_, are systematically higher than in girls belonging to analogical age groups (AG2–AG4). This discrepancy is because the absolute V̇_Emax_ (L min^−1^) values in the boys at a given age (above 10 years old) in boys are higher than in girls (see Table 5).

The presented low-cost particulate matter sensors allow for limiting the risk of health hazards in children by showing the actual PM concentrations and choosing the appropriate “time window” for the daily dose of exercise. The chosen period can be when air quality is the highest—in our research, the hours between 10 a.m. and 2 p.m. (see Fig. 2 and Table 4). In cases of heavy air pollution on a given day, teachers aware of this fact, might: perform their daily physical exercise inside the school or sports center buildings, or to limit the intensity and duration of outdoor exercise.

## Conclusions

The use of a low-cost measurement network^7^ supported by a calibration system^13^ is a useful tool in air quality monitoring, particularly in rural areas where the use of expensive, highly accurate measuring devices is beyond the budget of small communities. This low-cost measurement solution eliminates the limitations and social and informational exclusion affecting small communities such as villages (currently about 40% of the population in Poland)^9^. The presence of such installations in rural areas raises the awareness of residents regarding the role of air quality on their health and contributes to activating these communities for environmental protection. As shown in this study, the described low-cost particulate matter sensors monitoring the actual PM_10_ concentrations allow for limiting the risk of health hazards in children. This information enables school authorities and teachers to choose an appropriate “time window” for the daily dose of physical exercise performed outdoors when the air quality is the best to minimize the rate and total PM_10_ deposition in children’s respiratory tracts.

## Data Availability

The datasets used and/or analyzed during the current study available from the corresponding author on every request.

## References

[1] Ministry. *Regulation of the Minister of Climate and Environment of December 11, 2020 on assessing the levels of substances in the air***(in Polish)**. https://isap.sejm.gov.pl/isap.nsf/download.xsp/WDU20200002279/O/D20202279.pdf (2020).

[2] European Union. https://www.eea.europa.eu/publications/air-quality-in-europe-2021 (2021).

[3] WHO. *Global air quality guidelines. Particulate matter (PM2.5 and PM10), ozone, nitrogen dioxide, sulfur dioxide and carbon monoxide*. World Health Organization (2021). 34662007

[4] Saenen ND, Provost EB, Viaene MK, Vanpoucke C, Lefebvre W, Vrijens K, Roels HA, Nawrot TS (2016). Recent versus chronic exposure to particulate matter air pollution in association with neurobehavioral performance in a panel study of primary schoolchildren. Environ. Int..

[5] GOV. *Chief Inspectorate for Environmental Protection*. https://powietrze.gios.gov.pl

[6] Chen L-J, Ho Y-H, Lee H-C, Wu H-C, Liu H-M, Hsieh H-H, Huang Y-T, Lung S-CC (2017). An open framework for participatory PM2.5 monitoring in smart cities. IEEE Access.

[7] Nieckarz Z, Zoladz JA (2020). Low-cost air pollution monitoring system—An opportunity for reducing the health risk associated with physical activity in polluted air. PeerJ.

[8] Report. Air quality in Europe 2022. *European Environmental Agency*. 10.2800/488115 (2022).

[9] Yearbook. *Concise Statistical Yearbook of Poland*. https://www.stat.gov.pl (2021).

[10] Thangavel P, Park D, Lee YC (2022). Recent insights into particulate matter (PM2.5)-mediated toxicity in humans: An overview. Int. J. Environ. Res. Public Health.

[11] Chen G, Li S, Zhang Y, Zhang W, Li D, Wei X, He Y, Bell ML, Williams G, Marks GB, Jalaludin B, Abramson MJ, Guo Y (2017). Effects of ambient PM1 air pollution on daily emergency hospital visits in China: An epidemiological study. Lancet Planet. Health.

[12] Wilczyńska-Michalik W, Michalik M (2017). Air pollution in Krakow: A glance into the future from a historical perspective. Acta Geobalcanica.

[13] Nieckarz Z, Zoladz JA (2021). New calibration system for low-cost suspended particulate matter sensors with controlled air speed, temperature and humidity. Sensors.

[14] Lai N, Fiutem JJ, Pfaff N, Salvadego D, Strainic J (2021). Relating cardiorespiratory responses to work rate during incremental ramp exercise on treadmill in children and adolescents: Sex and age differences. Eur. J. Appl. Physiol..

[15] Reybrouck T, Weymans M, Stijns H, Knops J, van der Hauwaert L (1985). Ventilatory anaerobic threshold in healthy children. Age and sex differences. Eur. J. Appl. Physiol. Occup. Physiol..

[16] Rissler J, Nordin EZ, Eriksson AC, Nilsson PT, Frosch M, Sporre MK, Wierzbicka A, Svenningsson B, Löndahl J, Messing ME, Sjogren S, Hemmingsen JG, Loft S, Pagels JH, Swietlicki E (2014). Effective density and mixing state of aerosol particles in a near-traffic urban environment. Environ. Sci. Technol..

[17] Hussein T, Saleh SSA, dos Santos VN, Boor BE, Koivisto AJ, Löndahl J (2019). Regional inhaled deposited dose of urban aerosols in an eastern Mediterranean city. Atmosphere.

[18] Guo L, Johnson GR, Hofmann W, Wang H, Morawska L (2020). Deposition of ambient ultrafine particles in the respiratory tract of children: A novel experimental method and its application. J. Aerosol. Sci..

[19] Zoladz JA, Nieckarz Z (2021). Marathon race performance increases the amount of particulate matter deposited in the respiratory system of runners: An incentive for “clean air marathon runs”. PeerJ.

[20] *Physical Activity Guidelines for Americans* 2nd edn. https://health.gov/sites/default/files/2019-09/Physical_Activity_Guidelines_2nd_edition.pdf (2018).

[21] Rowland TW (2005). Children's Exercise Physiology.

[22] Mácek M, Vávra J, Novosadová J (1976). Prolonged exercise in prepubertal boys. I. Cardiovascular and metabolic adjustment. Eur. J. Appl. Physiol. Occup. Physiol..

[23] Astrand P-O, Rodahl K (1986). Textbook of Work Physiology.

[24] Wasserman K, Hansen JE, Sue DY, Stringer WW, Whipp BJ, Wasserman K, Hansen JE, Sue DY, Stringer WW, Whipp BJ (2005). Physiology of exercise. Principles of Exercise Testing and Interpretation.

[25] Zoladz JA, Szkutnik Z, Grassi B, Zoladz JA (2019). Metabolic Transitions and muscle metabolic stability: Effects of exercise training. Muscle and Exercise Physiology.

[26] Zoladz JA, Szkutnik Z, Majerczak J, Duda K (1998). Detection of the change point in oxygen uptake during an incremental exercise test using recursive residuals: Relationship to the plasma lactate accumulation and blood acid base balance. Eur. J. Appl. Physiol. Occup. Physiol..

[27] Zoladz JA, Duda K, Majerczak J (1998). Oxygen uptake does not increase linearly at high power outputs during incremental exercise test in humans. Eur. J. Appl. Physiol. Occup. Physiol..

[28] Canha N, Almeida M, do Freitas MC, Almeida SM, Wolterbeek HT (2011). Seasonal variation of total particulate matter and children respiratory diseases at Lisbon primary schools using passive methods. Proc. Environ. Sci..

[29] Löndahl J, Möller W, Pagels JH, Kreyling WG, Swietlicki E, Schmid O (2014). Measurement techniques for respiratory tract deposition of airborne nanoparticles: A critical review. J. Aerosol. Med. Pulm. Drug Deliv..

[30] Nunes RA, Branco PT, Alvim-Ferraz MC, Martins FG, Sousa SI (2015). Particulate matter in rural and urban nursery schools in Portugal. Environ. Pollut..

[31] Gautam S, Patra AK, Sahu SP, Hitch M (2016). Particulate matter pollution in opencast coal mining areas: a threat to human health and environment. Int. J. Min. Reclam. Environ..

[32] Hussein T, Al-Abdallat A, Saleh SSA, Al-Kloub M (2022). Estimation of the seasonal inhaled deposited dose of particulate matter in the respiratory system of urban individuals living in an Eastern Mediterranean City. Int. J. Environ. Res. Public Health.

[33] Zwozdziak A, Sówka I, Willak-Janc E, Zwozdziak J, Kwiecińska K, Balińska-Miśkiewicz W (2016). Influence of PM1 and PM2.5 on lung function parameters in healthy schoolchildren—A panel study. Environ. Sci. Pollut. Res.

[34] Ginsberg G, Foos B, Firestone M (2005). Review and analysis of inhalation dosimetry methods for application to children’s risk assessment. J. Toxicol. Environ. Health A.

[35] Morawska L, Afshari A, Bae GN, Buonanno G, Chao CYH, Hänninen O, Hofmann W, Isaxon C, Jayaratne ER, Pasanen P, Salthammer T, Waring M, Wierzbicka A (2013). Indoor aerosols: From personal exposure to risk assessment. Indoor Air.

